# Overexpression of CD163, CD204 and CD206 on Alveolar Macrophages in the Lungs of Patients with Severe Chronic Obstructive Pulmonary Disease

**DOI:** 10.1371/journal.pone.0087400

**Published:** 2014-01-30

**Authors:** Yoichiro Kaku, Haruki Imaoka, Yoshitaka Morimatsu, Yoshihiro Komohara, Koji Ohnishi, Hanako Oda, Shinichi Takenaka, Masanobu Matsuoka, Tomotaka Kawayama, Motohiro Takeya, Tomoaki Hoshino

**Affiliations:** 1 Division of Respirology, Neurology, and Rheumatology, Department of Medicine 1, Kurume University School of Medicine, Kurume, Fukuoka, Japan; 2 Department of Cell Pathology, Graduate School of Medical Sciences, Kumamoto University, Kumamoto, Japan; Helmholtz Zentrum München/Ludwig-Maximilians-University Munich, Germany

## Abstract

We have previously reported that the lungs of patients with very severe chronic obstructive pulmonary disease (COPD) contain significantly higher numbers of alveolar macrophages than those of non-smokers or smokers. M1 and M2 macrophages represent pro- and anti-inflammatory populations, respectively. However, the roles of M1 and M2 alveolar macrophages in COPD remain unclear. Immunohistochemical techniques were used to examine CD163, CD204 and CD206, as M2 markers, expressed on alveolar macrophages in the lungs of patients with mild to very severe COPD (Global Initiative for Chronic Obstructive Lung Disease (GOLD) stage I (mild) n = 11, II (moderate) n = 9, III (severe) n = 2, and IV (very severe) n = 16). Fifteen smokers and 10 non-smokers were also examined for comparison. There were significantly higher numbers of alveolar macrophages in COPD patients than in smokers and non-smokers. The numbers and percentages of CD163^+^, CD204^+^ or CD206^+^ alveolar macrophages in patients with COPD at GOLD stages III and IV were significantly higher than in those at GOLD stages I and II, and those in smokers and non-smokers. In patients with COPD, there was a significant negative correlation between the number of CD163^+^, CD204^+^ or CD206^+^ alveolar macrophages and the predicted forced expiratory volume in one second. Overexpression of CD163, CD204 and CD206 on lung alveolar macrophages may be involved in the pathogenesis of COPD.

## Introduction

Chronic obstructive pulmonary disease (COPD) is an important pulmonary inflammatory disease for which the prevalence and associated mortality rates have been predicted to rise. Smoking is recognized as the largest risk factor for COPD, and quitting smoking is thought to be important for prevention and control of COPD [1], [2]. However, there is no effective treatment for COPD-related pulmonary inflammation. We have previously demonstrated persistent and severe inflammation of small airways in the lungs of ex-smokers with very severe COPD. Furthermore the number of macrophages in the lungs of patients with very severe COPD was increased [3]. Increased numbers of CD8^+^ T-cells, alveolar macrophages and neutrophils are characteristic pathological features of the lungs in COPD [4], [5]. However, the effects of smoking on macrophage phenotypes in COPD are incompletely understood.

Macrophages display polarized phenotypes by which they can be divided into certain subpopulations. Proinflammatory or classically activated macrophages (M1) display pro-inflammatory and cytotoxic properties and can eradicate intracellular pathogens. In contrast, anti-inflammatory or alternatively activated macrophages (M2) display anti-inflammatory properties and are implicated in tissue repair [6], [7]. Granulocyte-macrophage colony stimulating factor (GM-CSF) and IFNs can generate M1 in vitro from human peripheral blood monocytes, and macrophage colony stimulating factor (M-CSF), IL-4 and IL-10 can generate M2 [8]. M1 macrophages secrete pro-inflammatory cytokines such as interleukin (IL)-12 and tumor necrosis factor (TNF)-α, have good antigen-presenting capacity, and promote Th1 immunity. In contrast, M2 macrophages secrete anti-inflammatory mediators such as IL-10, show poor antigen-presenting capacity, and promote the development of T-regulatory cells [8]–[10]. Alveolar macrophages show anti-inflammatory M2-characteristics [11]–[13], which can be distinguished from pro-inflammatory macrophages using M2 markers such as the scavenger receptors CD163 and CD204 [14], [15]. Although it has been unclear which phenotype has more phagocytic activity, the phagocytic capacity of alveolar macrophages is reported to be decreased in COPD patients who smoke, whereas it improves when patients quit smoking [16]. This suggests that a phenotypic alteration has occurred in COPD. Phenotypic changes in macrophages are considered to be involved in progression of diseases such as tumors [17], atherosclerosis [18] and renal disease [19].

The aim of the present study was to clarify the phenotypes of macrophages in the lungs of COPD patients in order to evaluate the role of macrophages in the pulmonary function of COPD patients.

## Materials and Methods

### Subjects

A total of 38 COPD patients (36 males and 2 females) were monitored at Kurume University Hospital (Kurume, Japan), Fukuoka University Hospital (Fukuoka, Japan). All were diagnosed on the basis of clinical history, physical examination, chest radiography, chest computed tomography and pulmonary function tests in accordance with the Global Initiative for Chronic Obstructive Lung Disease (GOLD) clinical criteria for the diagnosis and severity of COPD [20]. Exclusion criteria included chronic lung conditions such as asthma, bronchiectasis and interstitial lung disease, cardiac, hepatic or renal failure, and current oral steroid therapy. Lung tissues were obtained from 16 patients with very severe COPD (GOLD stage IV) who had undergone lung volume reduction surgery (LVRS) at Fukuoka University Hospital. Other lung samples were obtained from normal tissues around preserved cancer specimens that had been obtained surgically from 11 patients with mild COPD (GOLD stage I), 9 patients with moderate COPD (GOLD stage II), 2 patients with severe COPD (GOLD stage III), and 10 non-smokers and 15 smokers who had undergone resection of lung cancer at Kurume University Hospital. Lung diseases (e.g. sarcoidosis, infectious diseases) and collagen vascular diseases were carefully excluded from all subjects, and ex-smokers were carefully excluded from the group of non-smokers (Table 1). Informed written consent was obtained from all subjects, and sample collection and all procedures were approved by the ethics committees of Kurume University and Fukuoka University.

**Table 1:** Clinical characteristics of non-smokers, smokers and chronic obstructive pulmonary disease (COPD) patients examined by immunohistochemical analysis.

	Non-smoker	Smoker	COPD
** Patients**	10	15	38
** Age yrs**	63.6±8.8	64.7±4.8	68.7±5.2
**Sex**			
Male	7	6	36
Female	3	9	2
**GOLD stage**			
I (mild)	0	0	11
II (moderate)	0	0	9
III (severe)	0	0	2
IV (very severe)	0	0	16
**Smoking history**			
Current	0	15	8
Ex-smoker n (mean±sem yrs since smoking cessation)	0	0	30 (4.5±0.7)
Pack-yrs	0	37.0±7.8	27.7±6.1
** BMI kg/m^2^**	24.5±0.9	23.0±1.0	23.3±1.3
** %VC**	121.2±4.03	109.7±1.8	92.15±3.7
** %FEV_1.0_**	114.5±4.3	95.6±6.0	54.8±3.2
** FEV_1.0_/FVC%**	75.1±1.8	74.0±1.6	45.7±1.6
**Treatment**			
Systemic steroids	0	0	0
Inhaled corticosteroids	0	0	7
Bronchodilators	0	0	22(57.9)
No drug treatment	10(100)	15(100)	16(42.1)

Data are presented as n, n(%) or mean±SEM, unless otherwise stated. GOLD: Global Initiative for Chronic Obstructive Lung Disease; BMI: body mass index; VC: vital capacity; FVC: forced VC; % pred: % predicted; FEV_1.0_: forced expiratory volume in one second.

### Pulmonary Function Tests

Predicted normal Japanese values were used to calculate the vital capacity (VC), the forced vital capacity (FVC), the forced expiratory volume in one second (FEV_1.0_) and each % predicted, which met the Japanese Pulmonary Function Standard in the Japanese Respiratory Society Statement [21].

### Morphometric Analysis

The cross-sectional area occupied by the alveolar wall was quantified as the ratio of the total cross-sectional area, and the cross-sectional area occupied by the luminal mucosa was quantified as the ratio of the total cross-sectional area using a computer image analysis system, as reported by Hogg et al [22]. Digitized video images of the entire lung field were analyzed using a computerized color image analysis software system (Win Roof Version 5.0; Mitani Co., Fukui, Japan) as reported recently elsewhere [3], [23].

### Immunohistochemical Staining

For blockade of endogenous peroxidase activity, deparaffinized sections 3 µm thick were incubated with 1% H_2_O_2_ for 30 min. To detect macrophages, the tissues were reacted overnight at 4°C with anti-CD68 (ED1; Serotec, Oxford, UK), anti-CD163 (ED2; Serotec), anti-CD204 (SRA-E5; Transgenic, Kumamoto, Japan), anti-CD206 (Acris Antibodies, San Diego, CA), and anti-MMP-9 (Santa Cruz, Dallas, Texas). The samples were then washed extensively, and further incubated with appropriate horseradish peroxidase-conjugated secondary antibodies for 1 h at room temperature. After the removal of non-reacted secondary antibodies, the samples were incubated with 3,3′-diaminobenzidine-4HCl (DAB, Dako, Tokyo, Japan)-H_2_O_2_ solution to visualize the immunolabeling. Some sections were then counterstained with hematoxylin and eosin, and mounted with a coverslip. Double immunohistochemical analysis was performed as we previously reported [3]. CD163/CD204, CD163/CD206 and CD206/CD204 were double stained with DAB (brown) and HistoGreen (green) (Cosmobio, Tokyo, Japan), respectively.

### Quantitative Assessment of CD68^+^, CD163^+^, CD204^+^, and CD206^+^ Alveolar Macrophages in Lung Tissue

Quantitative assessment of macrophages was performed as reported previously with minor modification [3]. Briefly, immunostaining of CD68, CD163, CD204, and CD206 was performed using serial sections of lung tissues obtained from COPD patients who had undergone LVRS [24], [25]. Initially, nine square fields in which small-airway inflammation appeared most severe were selected. The numbers of CD68-positive cells, as alveolar macrophages (AMs), in the lung tissues were counted within these nine square fields and expressed as the number per mm^2^. The total numbers of AMs in non-smokers, smokers and COPD patients were expressed as mean ± SEM cells/mm^2^. Then CD163^+^, CD204^+^, and CD206^+^ cells were counted in the same fields as CD68^+^ cells. Two pathologists examined these sections independently in a blinded manner, without prior knowledge of the patients’ clinical status.

### Statistical Analysis

Results are expressed as means ± standard error of the mean (SEM). ANOVA was used to compare differences between groups. Nonparametric tests (Kruskal–Wallis and Mann–Whitney U-tests) were used to compare differences between the groups. Correlations were analyzed by simple regression. SAS 9.1.3 software, Japanese edition (SAS Institute, Cary, NC, USA), was used for statistical analysis. Differences at p<0.05 were considered to be statistically significant.

## Results

### Clinical Findings

Table 1 provides details (number, age, sex, GOLD stage, smoking history, body mass index, pulmonary function and treatment) of all individuals whose samples were subjected to immunohistochemical staining. All of the COPD patients who had undergone LVRS had stopped smoking 2–21years previously (mean 8.9±1.8 yr). Three COPD patients who had undergone LVRS had received inhaled beclomethasone dipropionate 400 µg/day and three were receiving 800 µg/day. Twenty-two COPD patients were receiving bronchodilators, such as β_2_-agonists, anticholinergics and/or methylxanthines. All the COPD patients analyzed were clinically stable and had experienced no disease exacerbations during the previous 3 months. We have analyzed the data to determine if there are any correlations between the number of alveolar macrophages and rate of exacerbation, infections and hospitalization. However there were no statistically significant correlations between those groups.

### Increased Number of CD68^+^ Alveolar Macrophages in Stage III/IV COPD Patients

**Figure 1:**
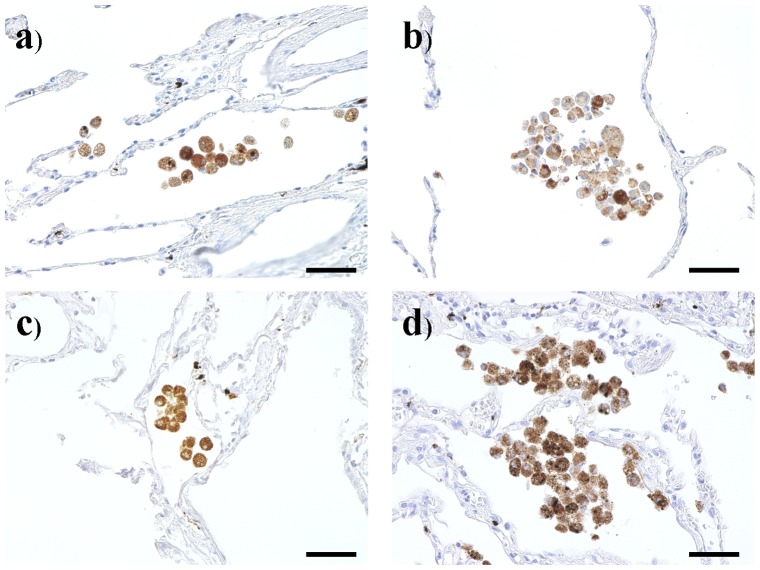
CD68 immunohistochemical staining of lung tissue samples from a) a non-smoker, b) a smoker, c) a mild chronic obstructive pulmonary disease patient (COPD), and d) a very severe COPD. Bar: 50 µm.

**Figure 2:**
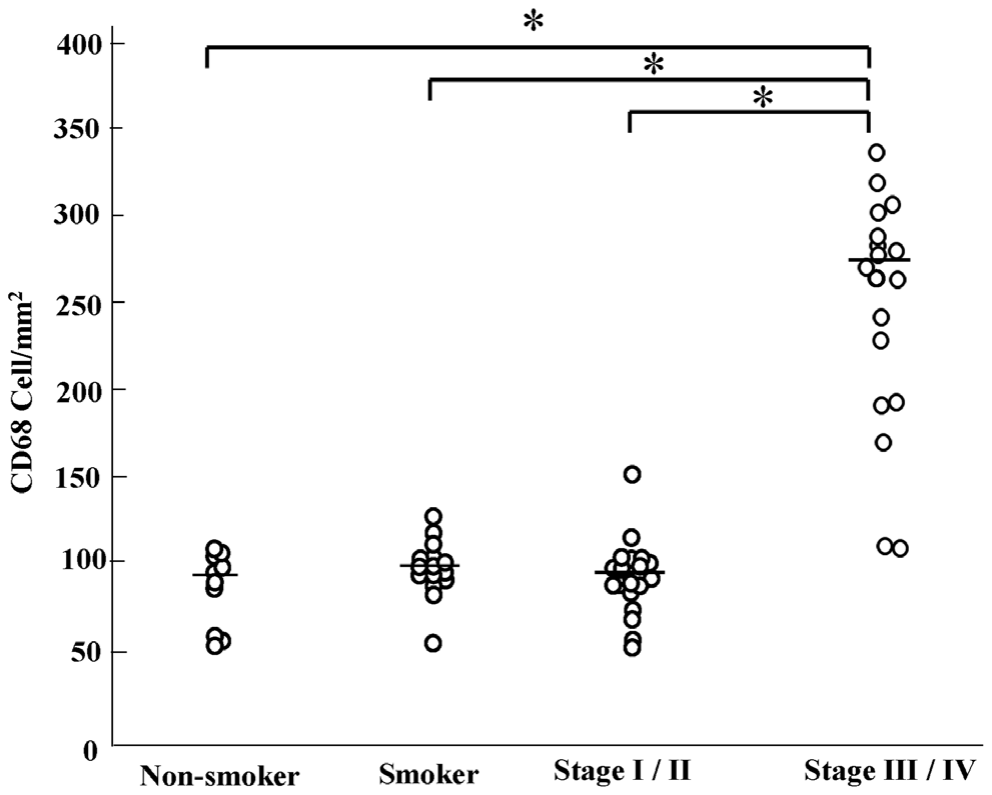
The number of CD68 positive cells in non-smoker, smoker and chronic obstructive pulmonary disease patient (COPD). *: p<0.01. The Global Initiative for Chronic Obstructive Lung Disease (GOLD) clinical criteria for the diagnosis and severity of COPD stage I: mild, II: moderate, III: severe, IV: very severe.

First, immunostaining for CD68 was performed using pathological sections to examine the distribution of all alveolar macrophages. As shown in Figs. 1 and 2, although CD68 staining intensity did not differ between non-smokers and smokers, or between different stages, the number of CD68^+^ alveolar macrophages was increased among stage III and stage IV patients (stage III/IV). Since there were no significant differences between the number of stage III and stage IV patients, and between the number of stage I and stage II patients, the patients with COPD were divided into two groups: stage I/II, and stage III/IV.

### Increased Numbers of CD163^+^, CD204^+^, and CD206^+^ Alveolar Macrophages in Stage III/IV COPD Patients

**Figure 3:**
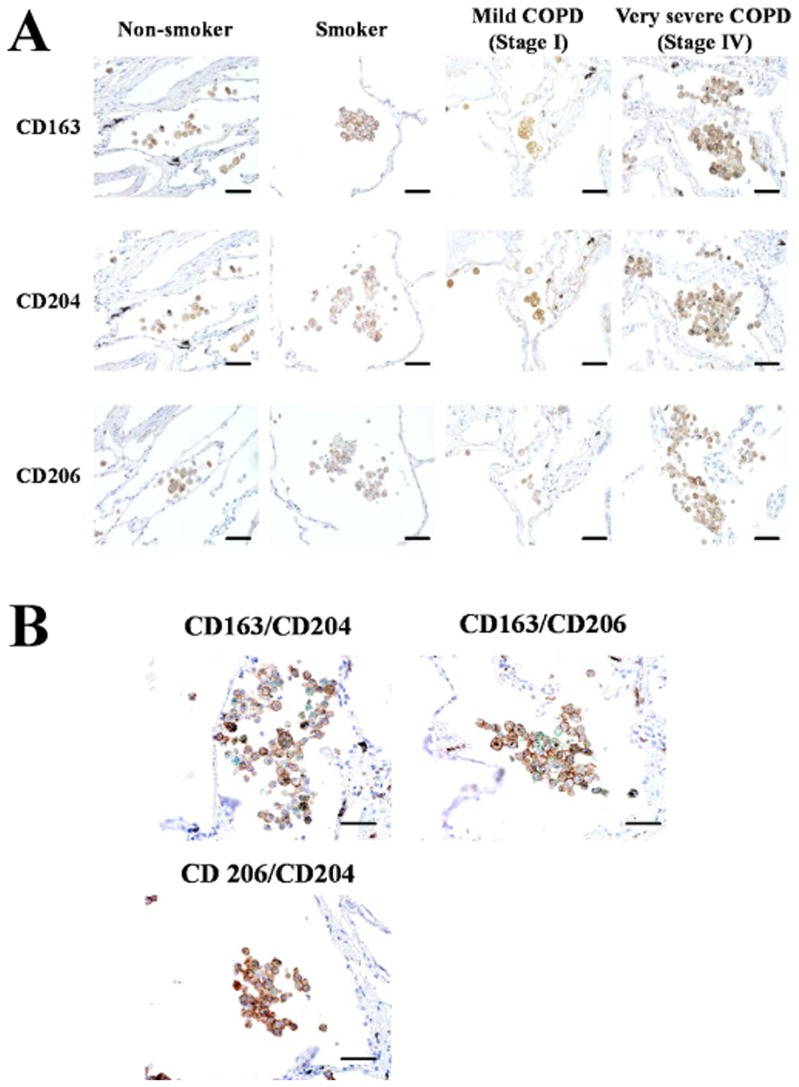
CD163, CD204 and CD206 immunohistochemical staining of lung tissue samples from a non-smoker, a smoker, a mild chronic obstructive pulmonary disease patient (COPD), and a very severe COPD. (A) Positive reactivity was identified by 3–3′-diaminobenzidine-4HCl (DAB). Bar: 50 µm. (B) Double immunohistochemical analysis for CD163/CD204, CD163/CD206 and CD206/CD204. Lung tissues were obtained from a very severe COPD. CD163 (brown)/CD204 (green), CD163 (brown)/CD206 (green) and CD206 (brown)/CD204 (green) were double stained with DAB (brown) and HistoGreen (green), respectively. Bar: 50 µm.

**Figure 4:**
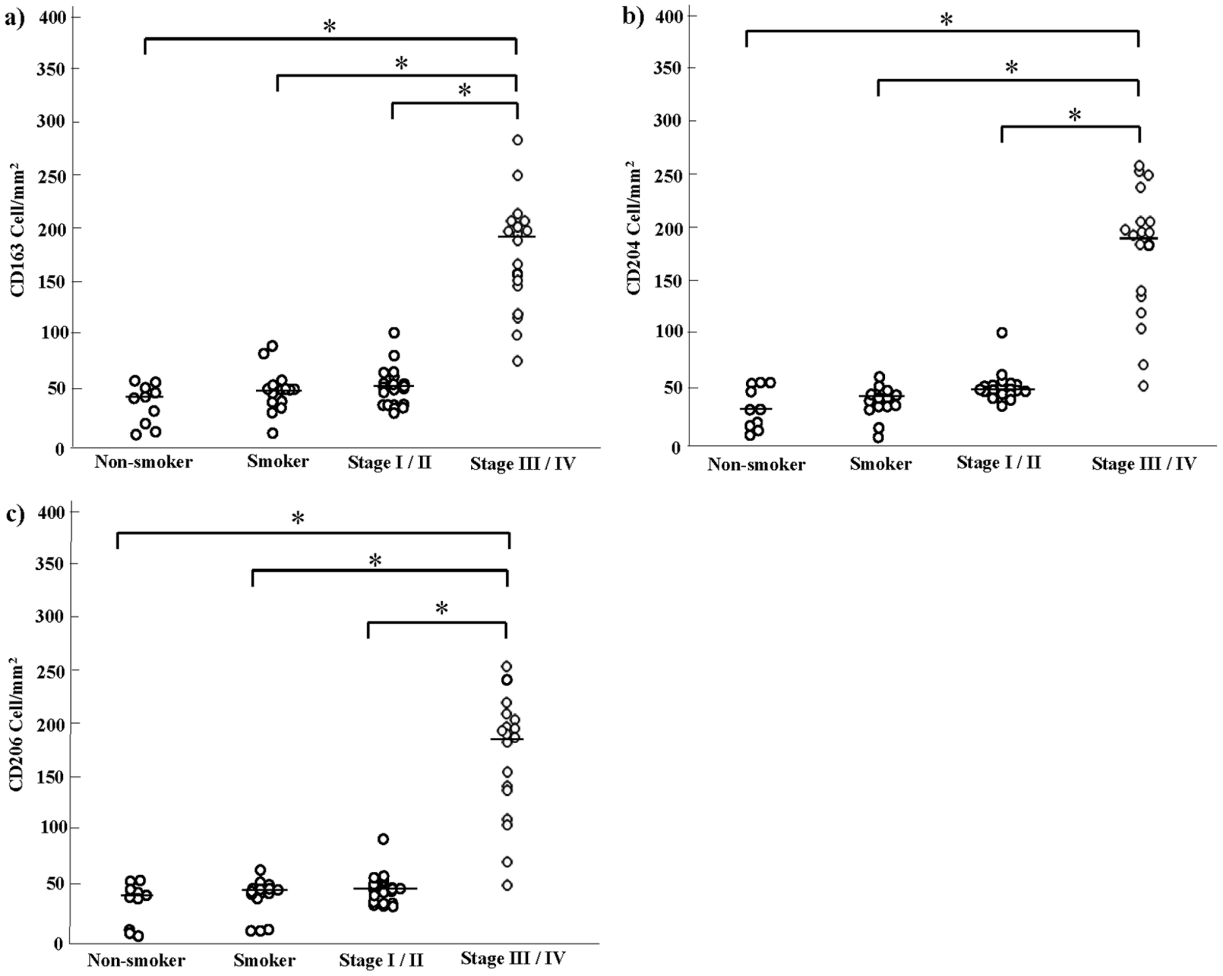
The number of CD163, CD204 and CD206 positive cells in non-smoker, smoker and chronic obstructive pulmonary disease patient (COPD). a) CD163, b) CD204, c) CD206, *: p<0.01. The Global Initiative for Chronic Obstructive Lung Disease (GOLD) clinical criteria for the diagnosis and severity of COPD stage I: mild, II: moderate, III: severe, IV: very severe.

It is well known that CD163, CD204, and CD206 are specifically expressed on macrophages and are useful as M2 macrophage markers. Therefore we performed immunostaining of CD163, CD204, and CD206 using serial sections. Similarly to the number of CD68^+^ macrophages, the numbers of macrophages positive for CD163, CD204, and CD206 were significantly increased in stage III/IV patients (Figs. 3 and 4). Double immunohistochemical analysis revealed that as shown CD163, CD204, and CD206 positive cells in stage III/IV COPD were increased (Fig. 3B).

### Increased Percentages of Cells Positive for CD163, CD204 or CD206 in the Lungs of COPD Patients Relative to Those in Non-smokers and Smokers

**Figure 5:**
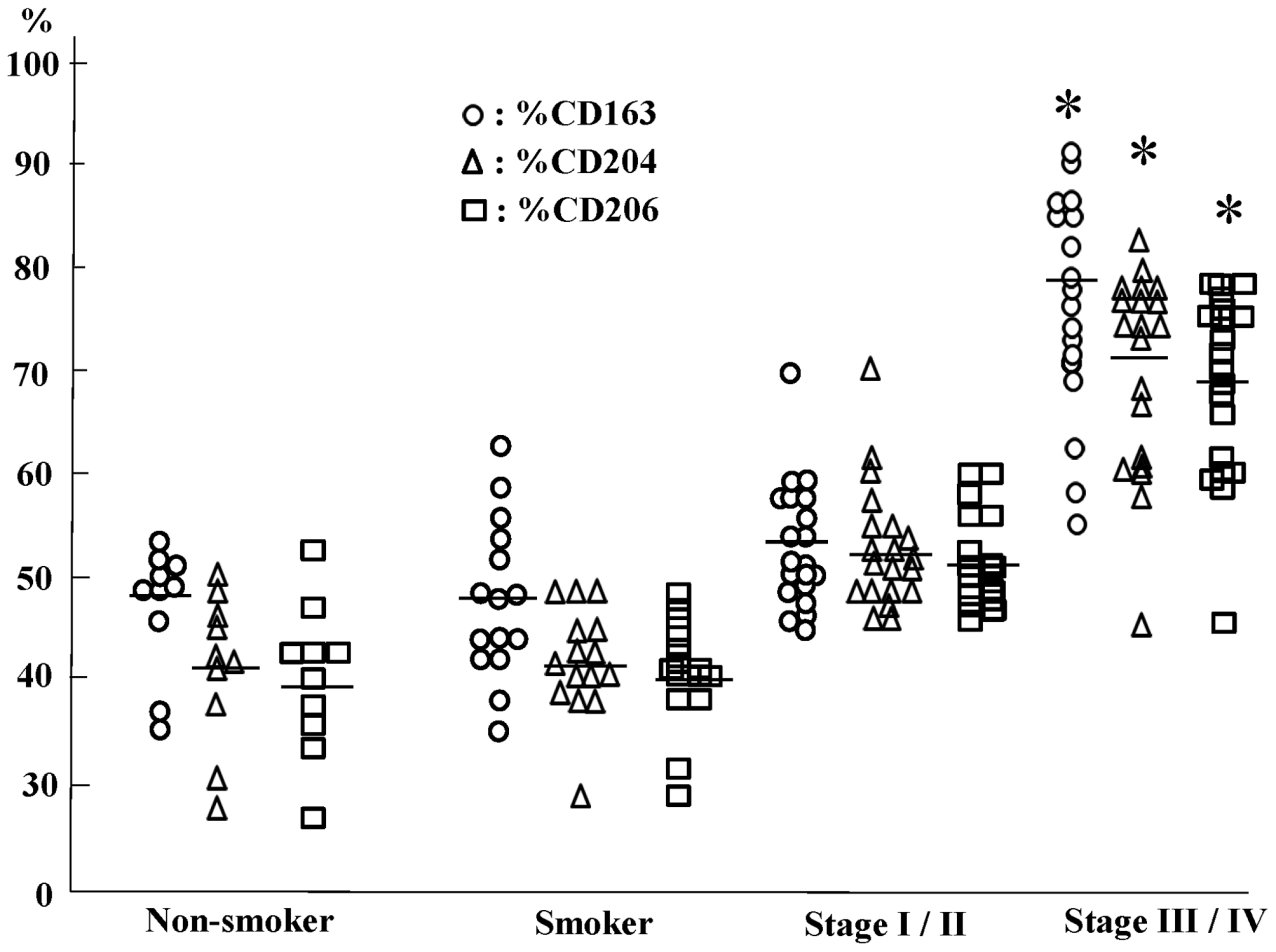
The percentage of CD163, CD204 and CD206 positive cell in non-smoker, smoker and chronic obstructive pulmonary disease patient (COPD). *: p<0.01 versus nonsmoker, smoker and stage I/II. The Global Initiative for Chronic Obstructive Lung Disease (GOLD) clinical criteria for the diagnosis and severity of COPD stage I: mild, II: moderate, III: severe, IV: very severe.

We then calculated the percentages of CD163^+^, CD204^+^, and CD206^+^ cells among CD68^+^ alveolar macrophages. CD163^+^, CD204^+^ and CD206^+^ cells accounted for approximately 40–45% of alveolar macrophages in the lungs of non-smokers and smokers, whereas they accounted for 80–70% of alveolar macrophages in patients with stage III/IV COPD. There were no significant differences in the percentages of CD163^+^, CD204^+^ or CD206^+^ cells between non-smokers and smokers or between non-smokers or smokers and COPD stage I/II patients (Fig. 5).

### Negative Correlation between the Numbers of Cells Positive for CD68, CD163, CD204 or CD206 and %FEV_1.0_ in COPD Patients

**Figure 6:**
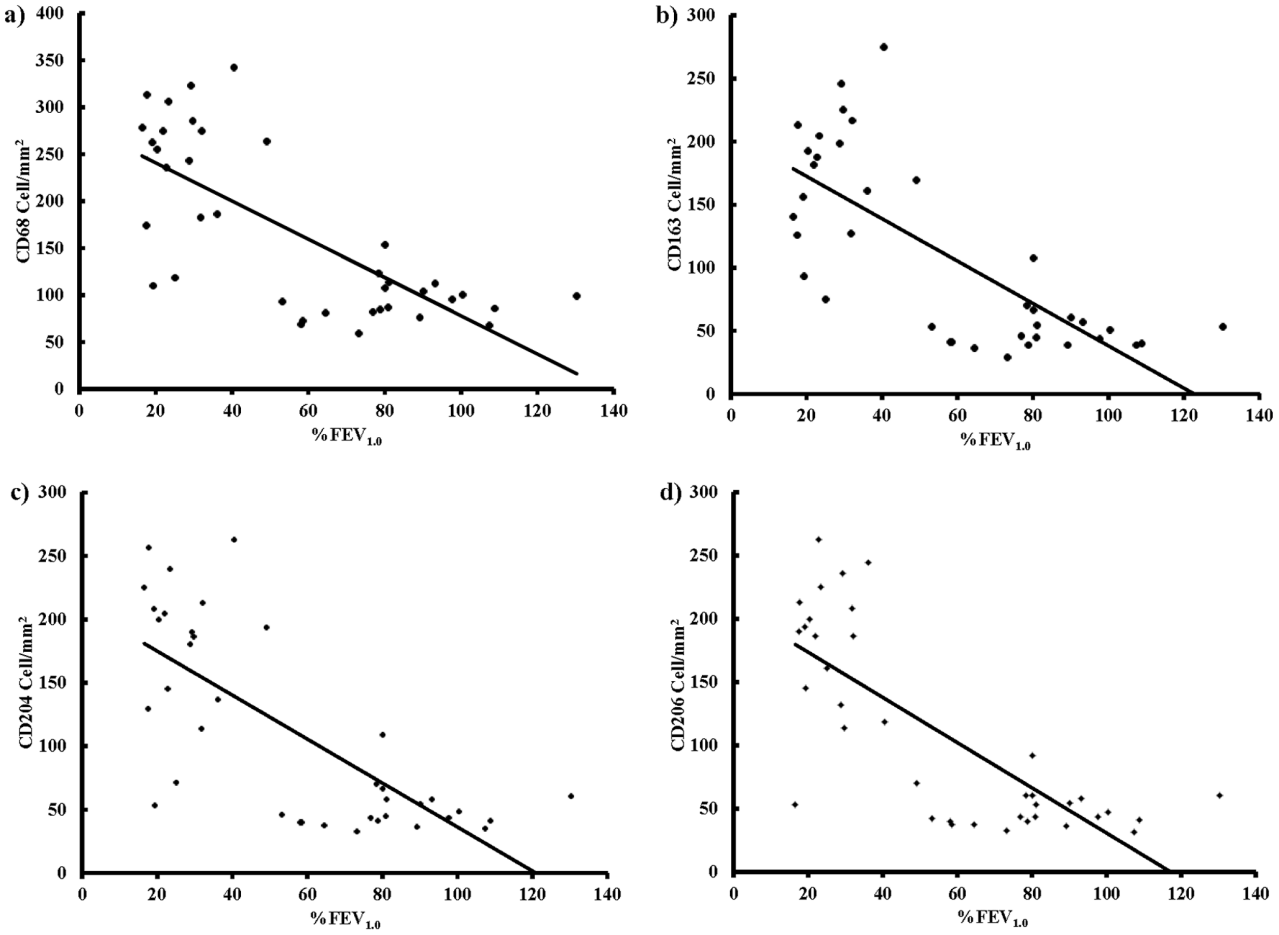
Correlation between the number of CD68, CD163, CD204 or CD206 positive cells and lung function measured as forced expiratory volume in one second % predicted (%FEV_1.0_) in chronic obstructive pulmonary disease patients (n = 38). a) The number of CD68 positive cells and %FEV_1.0_ (r = 0.729 and p<0.01), b) The number of CD163 positive cells and %FEV_1.0_ (r = 0.739 and p<0.01), c) The number of CD204 positive cells and %FEV_1.0_ (r = 0.732 and p<0.01), d) The number of CD206 positive cells and %FEV_1.0_ (r = 0.765 and p<0.01).

We also analyzed the correlation between the number of CD163^+^, CD204^+^ or CD206^+^ cells and pulmonary function in non-smokers, smokers and COPD patients. In COPD patients, there was a significant negative correlation between the number of CD163^+^, CD204^+^ or CD206^+^ cells and %FEV_1.0_, but not the FEV_1.0_/FVC% ratio (r = 0.729, r = 0.739, r = 0.732, r = 0.765; Fig. 6). In contrast, there were no significant correlations between the numbers of CD68^+^, CD163^+^, CD204^+^ or CD206^+^ cells and %FEV_1.0_ in non-smokers or smokers.

### MMP-9 Expressed in Alveolar Macrophages in the Lungs of COPD Patients

**Figure 7:**
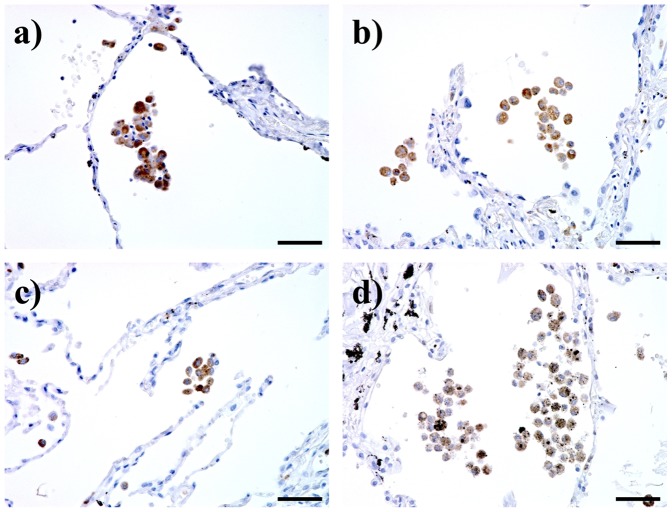
MMP-9 immunohistochemical staining of lung tissue samples from a) a non-smoker, b) a smoker, c) a mild chronic obstructive pulmonary disease patient (COPD), and d) a very severe COPD. Bar: 50 µm.

We analyzed the expression of matrix metalloproteinases (MMPs) in alveolar macrophages in the lungs of non-smokers, smokers and COPD patients by using immunohistochemical assay. MMP-9 was strongly expressed in alveolar macrophages in the lungs of non-smokers, smokers and COPD patients. Moreover, MMP-9 positive alveolar macrophages were increased in the lungs of very severe COPD (Fig. 7).

## Discussion

The present study demonstrated that CD163^+^, CD204^+^ and CD206^+^ macrophages were abundant in the lungs of ex-smokers with sever and very severe COPD (GOLD stage III/IV). In addition, there was a negative correlation between the number of CD163^+^, CD204^+^ or CD206^+^ cells and %FEV_1.0_ in COPD patients. These results suggest that cigarette smoke had activated alveolar macrophages and led to overexpression of CD163, CD204 and CD206. Both the lung tissues of non-smokers and smokers were obtained from the normal lung tissues derived from lung cancer patients. Thus, the effect of smoking versus non-smoking might be influenced by alveolar macrophage induction by the tumor environment. Further analysis is needed to test this possibility.

Previous studies have confirmed persistent and severe airway inflammation in lung tissues resected from ex-smokers with very severe COPD [26], [27]. Our previous results showed that cessation of smoking cannot prevent pulmonary inflammation in patients with severe COPD [3]. In this study, we found that the numbers of CD68-positive cells in the lungs of patients with severe and very severe COPD who had stopped smoking more than 2 years previously were significantly higher than in non-smokers, smokers and patients with mild or moderate COPD. Therefore, CD68-positive alveolar macrophages may be involved in the persistent and severe pulmonary inflammation seen in severe COPD.

The CD163, CD204, and CD206 antigens are known to be upregulated in M2-type macrophages, and are considered to be M2 markers [28], [29]. CD163 and CD204 (macrophage scavenger receptor class A) are receptors of the hemoglobin/haptoglobin complex and modified LDL, respectively, although recent studies have demonstrated that these receptors bind many ligands such as bacteria [30], [31]. CD206 (macrophage mannose receptor, C type I) is a receptor involved in phagocytosis of bacteria and fungi [32]. The mechanisms and roles of CD163 and CD206 in macrophage phenotypic changes have been unclear. Studies using CD204-deficient mice have shown that CD204 expression plays an important role in macrophage M2 polarization by inhibition of TLR signaling [33], [34]. Thomsen demonstrated that a variant of the CD204 gene, Arg293X, is related to increased risk of COPD [35]. Ohar demonstrated that CD204 overexpression induced by *MSR1* gene SNP is associated to high susceptibility to COPD [36]. These data suggest that CD204-induced M2 polarization of alveolar macrophages is involved in pathogenesis of COPD. M2 macrophages are known to preferentially secrete matrix metalloproteinases, which induce collagen destruction [37], [38]. Our data has shown that there are increased numbers of CD163^+^ CD204^+^ CD206^+^ M2 alveolar macrophages in stage III/IV COPD patients, and MMP-9 positive alveolar macrophages, presumably M2 macrophages, were increased in the lungs of very severe COPD. These findings suggest that mechanisms leading to accumulation of CD163^+^ CD204^+^ CD206^+^ M2 macrophages in the lung could contribute to severe emphysema and COPD. In this study, we divided COPD patients into two subgroups - into stages I/II and III/IV. The I/II group has 11 mild and 9 moderate patients while the III/IV group has 2 severe and 16 very severe patients. The difference in participant numbers in stage III (n = 2) and stage IV (n = 16) may influence the statistical evaluation of the results. Further analysis is needed to evaluate this issue. However, it has always been unclear which molecules are related to M2-induced COPD, and therefore further studies are necessary to clarify this association in more detail.

In this study, we showed that the numbers of M2 alveolar macrophages in stage III/IV COPD patients were significantly greater in non-smokers, smokers and stage I/II COPD patient. Our previous study has shown that IL-18 was strongly expressed in alveolar macrophages in the lungs of patients with very severe COPD [3]. These results suggest that overproduction of IL-18 from M2 alveolar macrophages in the lungs may be involved in the pathogenesis of COPD.

The treatment strategy for COPD consists mainly of bronchodilators, such as β2-agonists, theophylline and anticholinergics [20]. It is believed that there is no effective therapy for reducing the persistent pulmonary inflammation in patients with COPD and for improving their outcome, even in those who use inhaled corticosteroids [39], [40]. Therefore, the disease is being targeted with new anti-inflammatory treatments. The present study demonstrated macrophages showing overexpression of CD163, CD204 or CD206 in the lungs of patients with severe COPD. The present results raise the possibility that blockade of CD163^+^, CD204^+^ or CD206^+^ macrophages be a feasible treatment for COPD.
